# Peroneus brevis as source of instability in Jones fracture fixation

**DOI:** 10.1007/s00264-020-04581-2

**Published:** 2020-05-05

**Authors:** Madeleine Willegger, Emir Benca, Lena Hirtler, Lukas Moser, Shahin Zandieh, Reinhard Windhager, Reinhard Schuh

**Affiliations:** 1 Department of Orthopedics and Trauma Surgery, Division of Orthopedics, Medical University of Vienna, Waehringer Guertel 18-20, Vienna, 1090 Austria; 2 Center for Anatomy and Cell Biology, Division of Anatomy, Medical University of Vienna, Waehringer Straße 13, 1090 Vienna, Austria; 3 Department of Radiology and Nuclear Medicine, Hanusch Hospital, Vienna, Austria

**Keywords:** Jones fracture, Proximal fifth metatarsal, Peroneus brevis, Fracture-specific screw, Headless compression screw, Biomechanical

## Abstract

**Purpose:**

Intramedullary screw fixation is currently considered the gold standard treatment for Jones fractures in the athlete. Besides biological factors (i.e., poor vascularization), mechanical instability induced by the pull of the peroneus brevis tendon (PBT) contributes to deficient Jones fracture healing. This biomechanical study aimed to simulate loads induced by the PBT at the fifth metatarsal and to compare the stability of two intramedullary screw constructs in a Jones fracture fixation model.

**Methods:**

Jones fractures were created in 24 human paired specimens, and fixation was achieved with either a solid Jones fracture specific screw (JFXS) (Jones Screw; Arthrex Inc., Naples FL, USA) or a cannulated headless compression screw (HCS) (HCS; DePuySynthes, Solothurn, Switzerland). The PBT was fixed to a mechanical load frame by the use of a cryoclamp. Constructs were loaded in tension for 1000 cycles, followed by an ultimate load test. Construct failure was defined by exceeding 10° of dorsal angulation.

**Results:**

Preliminary failure occurred more often in HCS constructs (33%) compared to JFXS constructs (0%) (*P* = 0.044). Mean tensile load to failure reached 123.8 ± 91.4 N in the JFXS group and 91.5 ± 62.2 N in the HCS group (*P* = 0.337). The mean slope of the load-displacement curve was 24.2 ± 10.4 N/mm for JFXS constructs and 24.7 ± 5.5 N/mm for HCS constructs, respectively (*P* = 0.887).

**Conclusion:**

This is the first study evaluating the effect of PBT pull on the mechanical stability of Jones fracture fixation. Higher preliminary failure rates of HCS were found under cyclic loading conditions compared to JFXS.

**Electronic supplementary material:**

The online version of this article (10.1007/s00264-020-04581-2) contains supplementary material, which is available to authorized users.

## Introduction

Jones fractures and proximal fifth metatarsal stress fractures in zones II and III have a notoriously high rate of non-union and delayed union when treated conservatively [1–3]. Therefore, surgical fixation by the use of an intramedullary screw is currently recommended as primary treatment for highly active patients and athletes [4, 5]. In order to stabilize the fracture, the use of intramedullary screws dramatically improved union rates and enabled short recovery times with a fast return to sports [6–8]. An early mobilization and return to competition is desired, but without compromising the stability of fracture fixation. Impaired healing response can be attributed to a compromised blood supply at the metadiaphyseal region [9, 10], but mechanical instability may also play a detrimental role in Jones fracture healing [11–13]. It was postulated that early return to unrestricted sports activities might contribute to refracture development, screw failure, or non-union. Several reports of athletes sustaining a refracture on the first day of restarting sports activities caught the attention of treating surgeons, and raised the discussion about the ideal screw for stable fracture fixation [14, 15]. Cannulated screws are easy to implant, and headless compression screws might reduce post-operative screw head discomfort at the lateral foot [8]. Solid screws may sustain higher loads to failure, and solid fracture specific screws have been developed to better match the internal diameters of the fifth metatarsal [16, 17]. Nevertheless, clinical, randomized, controlled studies to assess the different efficacy of screws available are lacking.

The peroneus brevis muscle functions as evertor of the foot and as plantarflexor of the ankle. [18] The main function of the peroneal muscles (peroneus brevis and longus) during gait is to ensure mediolateral stability, and to prevent involuntary ankle inversion at foot strike. The activation of the peroneus brevis in normal walking is the greatest during single limb support, and in the push-off phase of the stride. [19–21] Since the peroneus brevis tendon (PBT) inserts at the proximal aspect of the fifth metatarsal, dynamic tensile forces act on the bone. Activation of the peroneus brevis during gait may potentially lead to displacement of the proximal fracture fragment in Zone II and III fractures, which are located distally to the PBT insertion. [1, 11, 18] Nevertheless, the role of the PBT as source of instability after Jones fracture fixation has been neglected so far. Early post-operative active mobilization may challenge the intramedullary stabilized Jones fracture by means of tensile forces which can lead to prolonged bone healing, or even result in early failure [11]. Reported biomechanical Jones fracture studies have used several test setups; however, intramedullary screw constructs have never been tested under dynamic loading of the PBT [22–28]. Since the type of screw for Jones fracture fixation is still matter of debate, a cannulated screw and a fracture-specific solid screw were compared using a novel biomechanical setup. This biomechanical study aimed to simulate peroneus brevis muscle pull exerted at the fifth metatarsal base comparing the stability of two intramedullary screw constructs in a laboratory Jones fracture fixation model. We hypothesized that a solid fracture-specific screw would show lower failure rates compared to a cannulated headless compression screw under dynamic and ultimate failure loading of the peroneus brevis tendon.

## Materials and methods

Twelve matched pairs of fresh human lower leg specimens disarticulated in the knee were used for this biomechanical study . The specimens were obtained from voluntary donors who consented to donate their body for research and teaching purposes to the Center for Anatomy and Cell Biology, Medical University of Vienna during the donor’s lifetime. The study was conducted in accordance with the guidelines of the 1964 Declaration of Helsinki and all subsequent revisions. The study was approved by the ethics committee of the Medical University of Vienna prior to conduction of the study (EK 2077/2013). Available specimens were included in this study if they were of sufficient soft tissue quality, free of evidence of previous trauma, or surgery at the mid- and hindfoot. Specimens with signs of vascular or neural problems in the foot region were also excluded. Additional exclusion criteria were a specimen age younger than 20, or older than 100 years. The specimens were stored at − 80 °C and thawed at + 4 °C 48 hours before study use in order to prevent tissue dehydration which can affect the tissue properties. Dual energy X-ray absorptiometry (DEXA) scans of the calcaneus were carried out to determine bone mineral density (BMD) before dissection and preparation of the specimens. BMD was assessed with Lunar, Prodigy series X-ray, and GE Medical Systems (GE Healthcare Europe GmbH, Vienna) and was reported as g/cm^2^.

**Fig. 1:**
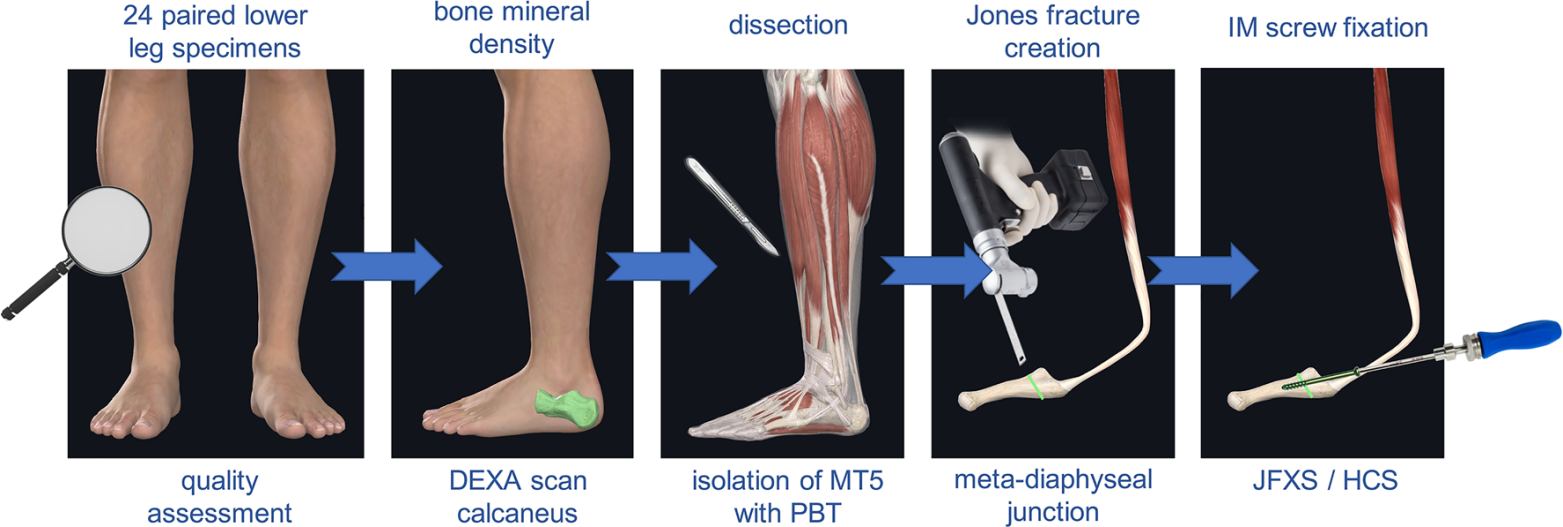
Specimen preparation scheme. Twenty-four paired lower leg specimens underwent quality assessment regarding inclusion and exclusion criteria. Dual energy X-ray absorptiometry (DEXA) scans of the calcaneus were carried out to determine bone mineral density (BMD) before dissection. The fifth metatarsal bone (MT5) was isolated under preservation of the peroneus brevis tendon (PBT) and muscle. A Jones fracture was created at the meta-diaphyseal junction at the distal aspect of the fourth tofifth intermetatarsal articular facet with an oscillating saw. Intramedullary (IM) screw fixation was performed with a solid Jones fracture specific screw (JFXS) in one group, and with a cannulated headless compression screw (HCS) in a second group.

### Specimen preparation

**Fig. 2:**
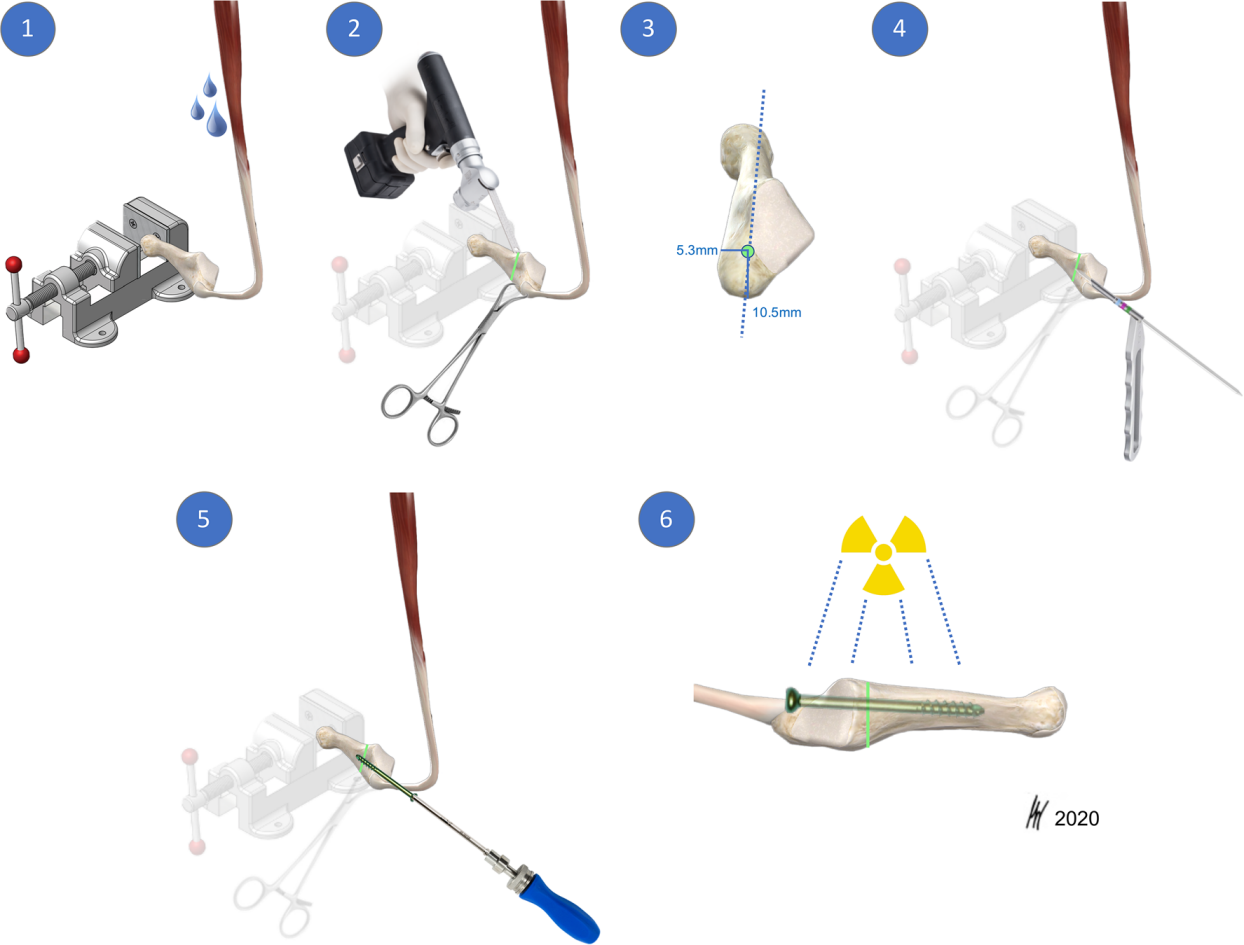
Intramedullary screw fixation technique. Intramedullary screw fixation is schematically outlined on a left fifth metatarsal using a 4.5-mm solid fracture specific screw (Jones fracture screw, Arthrex Inc., Naples, FL). In the first step (1), the fifth metatarsal bone was distally fixed in a padded machine vise. The bone and the peroneus brevis muscle were kept moist during preparation. (2) Then a complete Jones fracture at the meta-diaphyseal junction at the distal aspect of the 4^th^–5^th^ intermetatarsal articular facet (green line) was created by the use of an oscillating saw. A small forceps fixed and held the proximal fragment in place. In the next step, preparation for intramedullary screw fixation started by marking the entry point for the screw. (3) In a proximal dorsal view, the entry point is located “high and inside”, lateral to the cubometatarsal articular surface [5]. Anatomic studies described the ideal starting point 10.5-mm dorsal to the palpable inferior margin of the proximal tuberosity, and just medial to the peroneus brevis insertion. In a craniocaudal orientation the entry point is approximately 5.3-mm medial to the palpable lateral aspect of the tuberosity. [34] (4) A 2.0-mm guide wire is drilled into the metatarsal aiming for the straight part of the intramedullary canal. Afterwards, cannulated drilling with a 3.5-mm drill was performed followed by taping, starting with a 4.5-mm tap. The threads should tightly engage with the endosteal bone of the intramedullary canal. If the tap felt undersized, the next size was used (i.e., 6.0 for JFXS and 6.5 for HCS preparation). During taping, the appropriate screw size and length were measured by tactile feedback and by visual inspection superimposing the screw over the metatarsal. Screw length was determined as approximately 70% of the total length of the fifth metatarsal. Screw threads must cross the fracture line. (5) Finally, the guide wire was removed for solid screw (JFXS) insertion and the appropriate screw was inserted by hand. (6) Optimal screw size implantation was verified via X-ray control. Schematic view from medial.

The paired specimens (*n* = 24) originated from eight male and four female donors(Fig. 1). Mean donor age was 79 years (range 66–99). The peroneus brevis muscle was visualized through a lateral incision, then it was carefully dissected from its origin and followed distally to the insertion at the proximal aspect of the fifth metatarsal [18]. Care was taken to prepare the whole muscle belly with the intact tendon. The PBT insertion was not touched, and the fifth metatarsal was disarticulated from surrounding joints and liberated from soft tissue. Specimens were kept moist with saline solution to prevent tissue desiccation throughout the preparation process. Each specimen was screened for any evidence of previous open peroneal tendon surgery or Jones fracture fixation. Following inspection of the specimens, all pairs proved valid for the study and thereafter were assigned to two matched pair groups comprising an equal number of left and right feet for Jones fracture creation. The fifth metatarsal bone was distally fixed in a padded machine vise during fracture creation and preparation for intramedullary screw fixation (Fig. 2). A longitudinal line was drawn on the metatarsal in order to check for the rotational alignment. To simulate a Jones fracture (zone II), a complete transverse fracture at the meta-diaphyseal junction at the distal aspect of the fourth to fifth intermetatarsal articular facet was created with an oscillating saw. The proximal part of the bone was held in place with a small forceps during intramedullary screw preparation and placement. One group underwent intramedullary fixation with a Jones fracture-specific screw (Jones Screw; Arthrex Inc., Naples FL, USA) (JFXS group), while the other group was stabilized with a conventional cannulated headless compression screw (HCS; DePuySynthes, Solothurn, Switzerland) (HCS group). Screw size was determined upon the “fit and fill” principle. The intramedullary screw had to “fit and fill” the medullary canal with the threads across the fracture site. HCS were available in diameters 4.5-mm and 6.5-mm, and Jones Screws were used in diameters 4.5-mm and 6.0-mm. Small matched pair specimens (3/12) received 4.5-mm JFXS or HCS, and large specimen pairs (9/12) received 6.0-mm JFXS or 6.5-mm HCS.

### Biomechanical setup

**Fig. 3:**
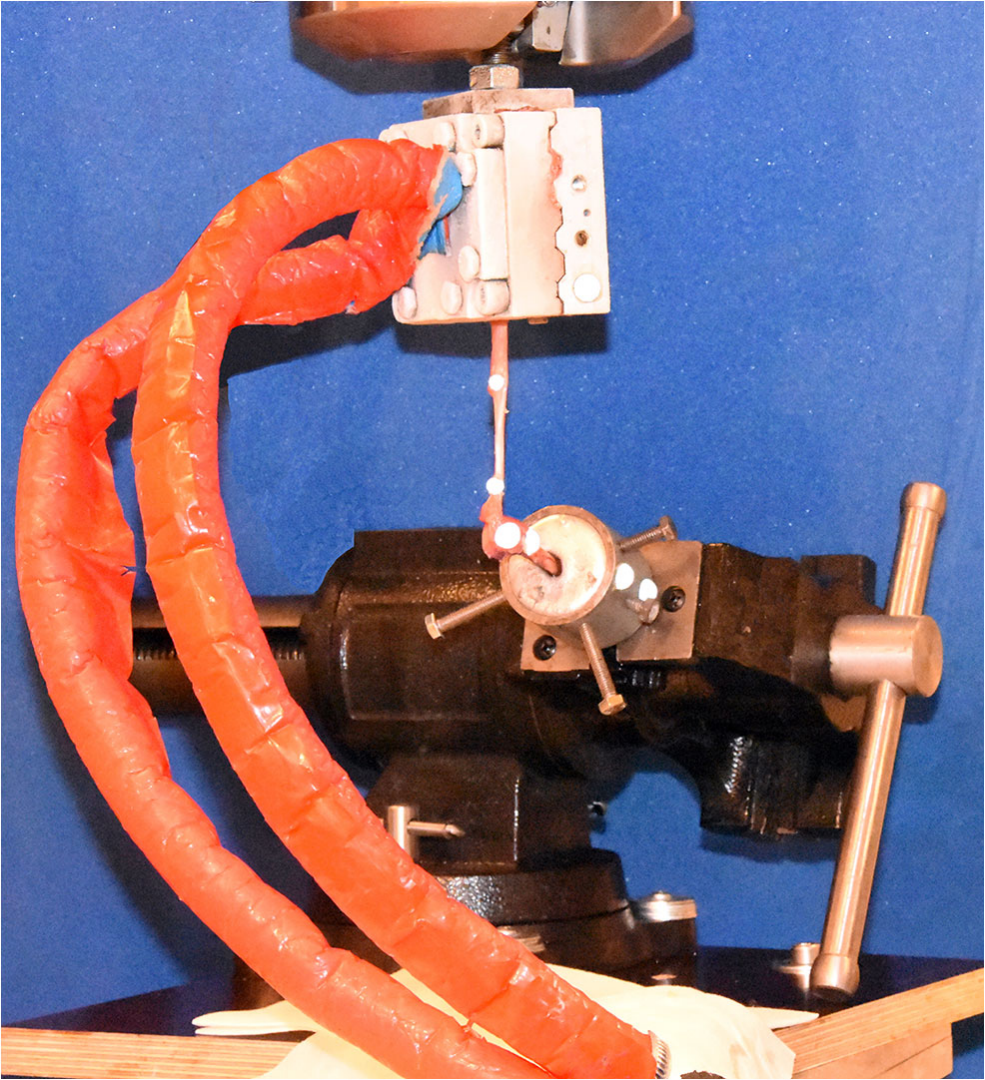
Biomechanical test setup. Light-reflecting markers were attached to the proximal aspect of the fifth metatarsal and to the fixation cup in order to record kinematic movements (angulation measurement). The distal aspect of the fifth metatarsal was fixed in a cup which was clamped in a machine vice. The peroneus brevis tendon was kept moist with saline solution until testing. The peroneus brevis muscle was fixated to the 858 Mini Bionix® (MTS® Systems Corporation) mechanical testing frame by the use of a cryoclamp. The construct was loaded in tension by the MTS testing machine.

A biomechanical setup was designed in order to simulate the pull of the peroneus brevis muscle (Fig. 3). We aimed to mimic tensile loads exerted by the peroneus brevis muscle. The fifth metatarsal specimens were potted with their distal aspects in Wood’s metal in 40-mm diameter custom built steel cups [29]. The steel cups were mounted in a machine vise on an adjustable platform. The fifth metatarsal was anatomically aligned in slight plantarflexion of 7–10°. A conventional self-leveling horizontal laser beam (PLL 360; Robert Bosch GmbH, Leinfelden-Echterdingen, Germany) and a goniometer were used to align the specimens during mounting. The muscle belly of the peroneus brevis was clamped 7-cm proximal to its insertion in a custom-designed cryo-fixation clamp with sawtooth grips to prevent slippage of the tendon during testing [30]. The cryo-clamp was connected to the actuator of a biaxial mechanical load frame (858 Mini Bionix, MTS Systems Corporation, Eden Prairie, MN, USA). Two 5-mm reflecting hemispherical markers glued to the midportion of the tendon additionally monitored slippage and tendon elongation during testing. The maximum temperature of −15°C of the cryo-fixation clamp was monitored steadily at the inserting site using a conventional laser thermometer. The interfragmentary angulation between the proximal and the distal aspect of the Jones fracture specimen was measured using an opto-electronic motion capture system (Smart-E; BTS Bioengineering, Milan, Italy) with four cameras with a sampling rate of 120 Hz. Two 5-mm reflecting hemispherical markers were attached to the two segments using epoxy glue. The force measurement transducer was integrated into the 858 Mini Bionix® testing system with a reported uncertainty of 1%. Video recording (D7200; Nikon, Tokyo, Japan) of each specimen was performed during the testing process (Supplementary Video 1).

### Cyclic and ultimate load testing

The biomechanical test protocol started with a preloading of the construct for two seconds to eliminate slackness between the specimen and the test setup, followed by a relaxation to 0 N. Each specimen was loaded subsequently for 1000 cycles followed by a load to failure test. Tensile load was applied in line with the peroneus brevis tendon with a peak load of 25 N at a rate of 3 N/s. Before unloading at the same rate, this position was held for five seconds. The loading amplitude ranged from approximately 5 to 25 N and it was applied in a force-controlled manner. The peak load magnitude was 1/3 of the mean failure load of intramedullary screw fixation in a fifth metatarsal tuberosity fracture fixation model, determined by Moshirfar et al. [13], and was selected to substantially challenge the construct without prematurely damaging it during cyclic loading. Load was recorded continuously at a sampling rate of 60 Hz during testing. The cyclic load mean reached 10.10 N at 0.5 Hz. Axial displacement was recorded at cyclic load segments: 1, 10, 100, 200, 300, 400, 500, 600, 700, 800, 900, and 1000. After completion of loading cycle 1000, a subsequent quasi-static load to failure test at a rate of 0.1 mm/s was carried out without unloading the construct. Load-displacement curves for each construct were filtered using the digital Savitzky-Golay filter before analysis. The ascending linear region of the load-displacement curve was used to measure the slope (N/mm). We used the term “slope of the load-displacement curve” instead of stiffness, as frequently found in the literature [29]. Fracture site angulation was measured during the whole testing protocol. Failure of the construct was defined by an interfragmentary angulation of > 10° or by gross failure (i.e., fracture). In constructs that failed preliminarily, the maximum load applied at the time of preliminary failure was defined as tensile load to failure. Failure modes were classified according to video analysis and direct visual inspection of the constructs by two orthopaedic surgeons (MW, RS).

### Statistical analysis

Statistical analyses were performed using SPSS 25 for Mac (SPSS Inc., Chicago, IL, USA), and the level of significance was set at the 95% confidence level (CI), with *P* values of < 0.05. Descriptive data were reported as means with range and standard deviation (SD). All data showed a normal distribution in Kolmogorov-Smirnov test. The independent *t* test was used to determine the significance of differences in slope, angulation, and tensile load to failure between the experimental groups. Intergroup comparison of nominal data was carried out by the use of the Pearsons Chi-Squared Test.

## Results

One construct had to be excluded from analysis due to a mechanical testing machine error (JFXS construct). In another specimen, loosening at the steel cup-machine vice-interface occurred during load to failure testing and therefore it had to be excluded from analysis (JFXS construct). In total, 22 specimens completed the testing protocol and were included for analysis. There was a statistically significant difference in BMD between male and female specimens. The mean BMD in male specimens reached 0.551 g/cm^2^ (range 0.312–0.915) compared to 0.302 g/cm^2^ (range 0.023–0.625) in female specimens (*P* = 0.012, 95% CI 0.06 to 0.44). The BMD was similar among the groups (JFXS group: mean 0.434 g/cm^2^, range 0.023–0.800; HCS group: mean 0.483 g/cm^2^, range 0.179–0.915, *P* = 0.634, 95% CI − 0.26 to 0.16).

### Preliminary failure and angulation

Four constructs failed during cyclic loading by exceeding 10° of dorsal angulation. Two specimens failed between the first and tenth cycle, another one failed between the tenth and 100^th^ cycles, and another one failed between the 700^th^ and 800^th^ cycles, respectively. All preliminary failures occurred in the HCS group. This difference in preliminary failure rates was statistically significant between the screw groups (*P* = 0.044) (Tab. 1). Initial angulation at the first loading cycle reached 2.1 ± 0.9° in the JFXS group and 3.8 ± 3.1° in the HCS group (*P* = 0.289, 95% CI − 4.79 to 1.55). At cycle 1000, angulations of the fracture fragments were 4.0 ± 1.5° and 4.2 ± 1.8°, respectively (*P* = 0.861, 95% CI − 2.45 to 2.09) (Tab. 2).

**Table 1:** Preliminary failure during cyclic loading

Group	JFXS (*n* = 10)	HCS (*n* = 12)	*P* value
Preliminary failure	0 (0%)	4 (33.3%)	0.044

**Table 2:** Construct survival and angulation

Cycle		1	10	100	200	300	400	500	600	700	800	900	1000
JFXS (*n* = 10)	*n*	10	10	10	10	10	10	10	10	10	10	10	10
	Mean (°)	2.1	2.7	3.2	4.5	3.5	3.6	3.7	4.5	4.7	3.8	3.9	4.0
	SD	0.9	1.0	1.3	2.9	1.6	1.6	1.7	2.6	2.7	1.7	1.7	1.5
HCS (*n* = 12)	*n*	12	10	9	9	9	9	9	9	9	8	8	8
	Mean (°)	3.8	2.8	3.2	3.5	3.6	3.7	3.9	4.0	4.1	4.0	4.0	4.2
	SD	3.1	1.6	1.5	1.5	1.6	1.6	1.6	1.6	1.7	1.7	1.8	1.8

### Tensile load to failure and slope

**Fig. 4:**
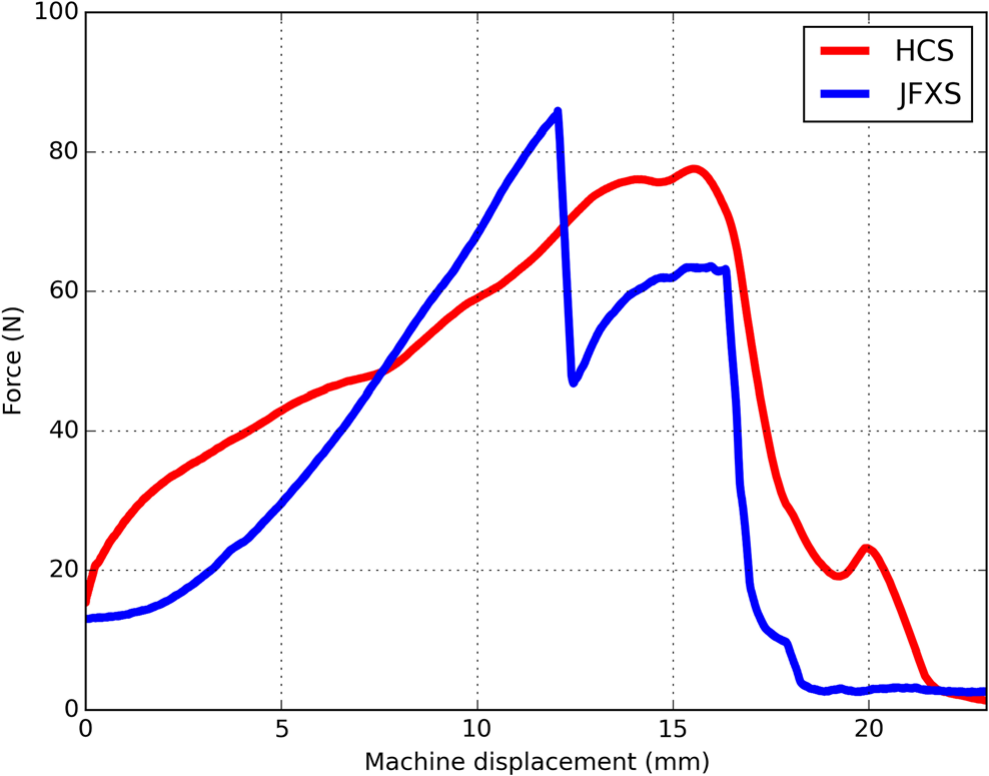
Load-displacement curves Representative load-displacement curves for two paired specimens. Red: headless compression screw (HCS) construct; Blue: Jones fracture-specific screw (JFXS) construct

Mean tensile load to failure reached 123.8 ± 91.4 N (range 48.1–370.7) in the JFXS group and 91.5 ± 62.2 N (range 37.9–209.4) in the HCS group (*P* = 0.337, 95% CI − 36.17 to 100.81). The mean slope of the load-displacement curves in load to failure tests for the JFXS constructs was 24.2 ± 10.4 N/mm (range 9.2–40.5) and 24.7 ± 5.5 N/mm (range 17.6–33.3) for the HCS constructs, respectively (*P* = 0.887, 95% CI − 8.34 to 7.28) (Fig. 4).

### Mode of failure

**Fig. 5:**
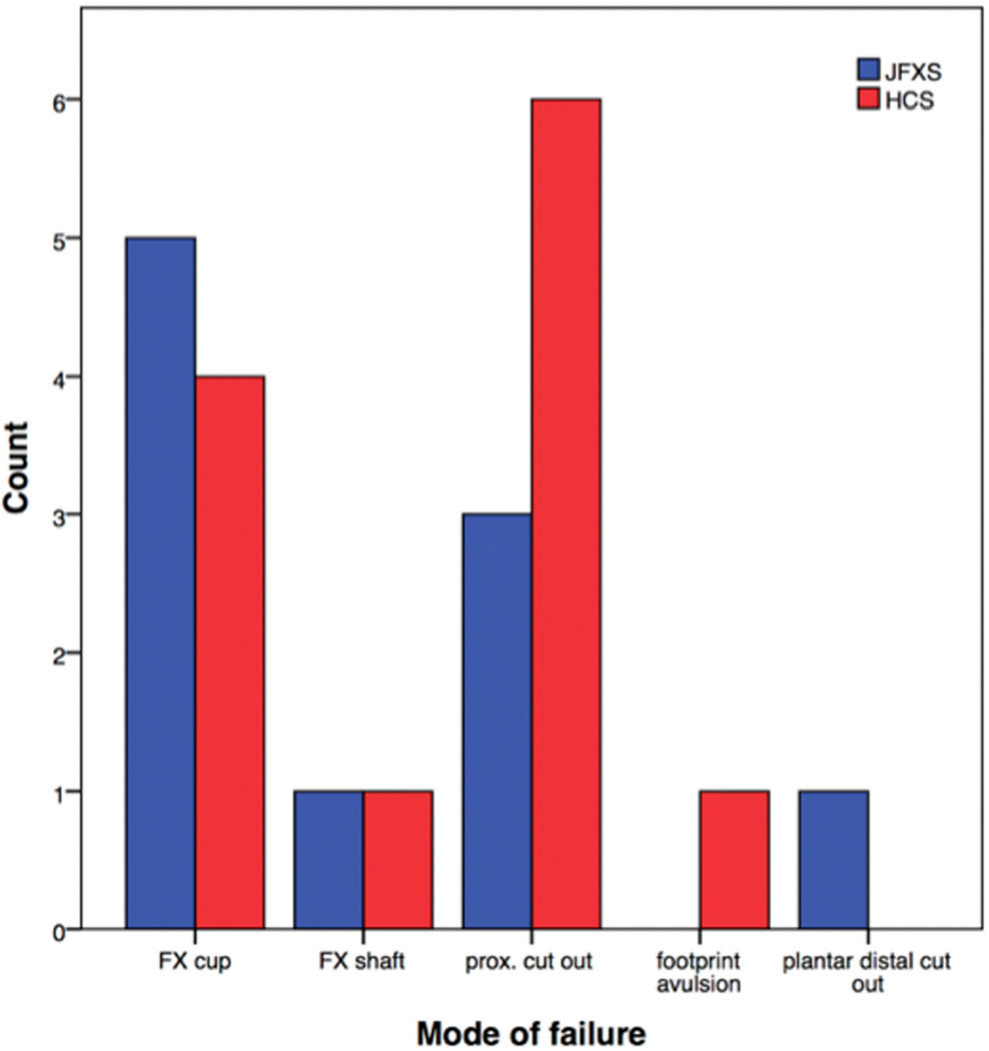
Mode of failure distribution FX cup is the metatarsal fracture at the steel cup; FS shaft is the metatarsal fracture at the shaft; prox. cut out is the cut out of the screw head at the proximal aspect of the metatarsal; footprint avulsion is the bony avulsion of the peroneus brevis footprint; plantar distal cut out is the cut out of the screw threads at the plantar distal shaft

Bony cut out of the proximal screw head was the most common mode of failure among HCS constructs (*n* = 6; 50%), followed by fracture of the metatarsal at the level of embedding in the steel cup (*n* = 4; 33%) (Supplementary Video 1). In JFXS constructs, the most common mode of failure was classified as fracture of the metatarsal at the steel cup (*n* = 5; 50%) followed by a bony cut out of the screw head (*n* = 3; 30%) (Fig. 5). No screw bending or screw breakage was observed.

## Discussion

The role of the PBT as a source of biomechanical instability on proximal fifth metatarsal fractures has been under-estimated and under-represented in biomechanical studies to date. Early mobilization and increase in sports activities before complete bony union may induce excessive loads on the stabilized fifth metatarsal by a combination of plantar loading, shear forces, and tensile forces [12]. The influence of these mechanical characteristics on Jones fracture healing remains still unknown. Nevertheless, impaired initial post-operative stability and early active mobilization can lead to failure after intramedullary screw fixation [3, 6, 14]. We aimed to design a test setup to simulate peroneus brevis muscle activity and pull at the fifth metatarsal base in order to test the construct stability of two intramedullary screw types.

We found a mean tensile load to failure of 123.8 ± 91.4 N in the JFXS group and of 91.5 ± 62.2 N in the HCS group (*P* = 0.337). The mean slope of the load-displacement curves reached 24.2 ± 10.4 N/mm and in the JFXS and 24.7 ± 5.5 N/mm in the HCS constructs, respectively (*P* = 0.887). The differences between the construct groups were not statistically significantly different.

Preliminary failure during cyclic tension loading occurred significantly more often in HCS constructs (33%) than in JFXS constructs (0%) (*P* = 0.044). Regarding the mode of failure, the countersunk head of the screw played a major role. Half of the HCS specimens failed due to a cut out of the screw head. This mode of failure occurred more often in the headless compression screw constructs. We hypothesize that the engaging threads of the countersunk head of the HCS cuts through the cancellous bone in the metaphysis during loading and therefore the screw loosens more easily. Another common failure mode in both construct groups was a fracture of the metatarsal shaft, just at the distal aspect, where the metatarsal was embedded in the steel cup. This mechanism might be owed to the test setup and is not a typical mechanism of failure in vivo. Nevertheless, this failure shows that fixation of the metatarsal in the cup was sufficient to detect bone or screw failure. The area of the metatarsal between intramedullary fixation and potting represents the weakest area with the highest lever forces in the test setup.

Most biomechanical Jones fracture studies focused on three-point bending tests, plantar to dorsal loading mechanisms, pull-out tests, and torsional forces acting on the fracture site. [16, 22–28] Three-point bending test and pull-out do not accurately replicate the type of loads that the screw and bone construct would experience during post-operative weight-bearing, as these tests represent a worst-case loading situation. Dorsal to plantar loading test setups however simulate loading conditions on the metatarsal during walking, but neglect forces which are produced by muscle pull. We are not aware of any study which performed cyclic loading of the peroneus brevis tendon in a Jones fracture fixation model. Nevertheless, there is no doubt that muscle pull (i.e., pull of the peroneus brevis tendon) causes tensile forces on the proximal fracture fragment since tension band wiring has been proven to work in Jones fracture fixation [31]. In intramedullary screw fixation, pull of the PBT might play a role in producing instability and micromotion at the fracture site, but only a paucity of studies even discuss PBT pull and its involvement in fifth metatarsal fractures [11–13, 32]. Morris et al. [11] evaluated the mechanical effect of the PBT on fifth metatarsal avulsion (zone I) and metadiaphyseal fractures (zone II), investigating the tendon as a source of biomechanical instability. They found that proximal fifth metatarsal fractures distal to the PB insertion were significantly more unstable than more proximal fractures. In our study, all fractures were created distal to the PBT insertion mimicking maximum instability due to PBT pull. Moshirfar et al. [13] performed load to failure tests on simulated tuberosity avulsion fractures (zone I) that were fixed with either an intramedullary screw or with a bicortical lag screw. The test setup consisted of a servohydraulic testing machine and a clamp which fixated the PBT tendon. The distal aspect of the fifth metatarsal was potted. In the present study, the setup was chosen based on this previously published study with few adaptations regarding the specimen potting and the loading conditions.

This study has to face several limitations. First, the number of tested specimens was relatively low and donor age was high, which is common in biomechanical studies due to unavailability of human anatomical specimens. Second, the loads for cyclic loading were chosen arbitrarily [13]. Little is known concerning the magnitude of loads exerted by the PBT to the fifth metatarsal base during walking, running, or jumping [33]. Nevertheless, we believe our model is a reasonably accurate estimation of loading conditions exerted by the peroneus brevis tendon to the base of the fifth metatarsal. The use of a dynamic gait simulator model could help to combine plantar loading, tensile, and torsional forces in one test setup. Third, a biomechanical study can never give a true representation of fracture pattern, in vivo failure, or account for fracture healing. However, observation of the mode of failure of the HCS in the present study showed a consistent pattern with previously published data in terms of a bony cut out of the threads engaged at the proximal fifth metatarsal [22]. Fourth, we had to face technical problems with the cup fixation in the machine vice in one specimen and a mechanical testing machine error in another specimen. Due to the exclusion of these two specimens, a paired statistical analysis was unfortunately not reasonable. Fifth, we used 4.5-mm JFXS or HCS in smaller specimens and compared a 6.0-mm JFXS to a 6.5-mm HCS fixation in lager specimens, in order to compare equal screw sizes. The intermediate screw size of 5.5 mm, which is available for the Jones fracture specific screw type only, was not used. These limitations should be kept in mind when interpreting the results.

In athletes who sustain a Jones fracture, intramedullary screw fixation is indicated in order to allow for an early regimen, and to enable a return to sports as fast as possible [6–8]. Intramedullary screw fixation should withstand bending, torsion, as well as tension. In clinical studies, there is no consensus about the ideal screw type to use [17]. Biomechanical studies can potentially shed some light on the initial stability provided by different screw types. Even though the results of this experimental study cannot be transferred directly to the clinic, higher preliminary failure rates of headless compression screws in comparison to solid fracture specific screws during cyclic loading theoretically speak against the use of HCS screws treating an athlete followed by an early active rehabilitation protocol, which typically involves gentle, cyclic range of motion exercises. Since there is no available data regarding failure rates, complications, and return to sports time comparing the use of cannulated headless compression screws, or solid fracture-specific screws for Jones fracture fixation, further clinical studies are needed to prove the biomechanical findings.

In conclusion, higher preliminary failure rates of headless compression screws in comparison to solid fracture specific screws have been found in a simulated Jones fracture model under cyclic loading of the peroneus brevis tendon.

## Electronic supplementary material

ESM 1 (MP4 13,439 kb)
